# Autism spectrum disorder at the crossroad between genes and environment: contributions, convergences, and interactions in ASD developmental pathophysiology

**DOI:** 10.1186/s13229-020-00370-1

**Published:** 2020-09-10

**Authors:** Cristina Cheroni, Nicolò Caporale, Giuseppe Testa

**Affiliations:** 1 High Definition Disease Modelling Lab, Stem Cell and Organoid Epigenetics, IEO, European Institute of Oncology, IRCCS, Milan, Italy; 2 Department of Oncology and Hemato-oncology, University of Milan, Milan, Italy; 3 Human Technopole, Via Cristina Belgioioso 171, Milan, Italy

**Keywords:** Autism spectrum disorder, Neurodevelopmental disorders, Pluripotent stem cells, Brain organoids, Developmental neurotoxicology, Endocrine disruptors, Gene × environment

## Abstract

The complex pathophysiology of autism spectrum disorder encompasses interactions between genetic and environmental factors. On the one hand, hundreds of genes, converging at the functional level on selective biological domains such as epigenetic regulation and synaptic function, have been identified to be either causative or risk factors of autism. On the other hand, exposure to chemicals that are widespread in the environment, such as endocrine disruptors, has been associated with adverse effects on human health, including neurodevelopmental disorders. Interestingly, experimental results suggest an overlap in the regulatory pathways perturbed by genetic mutations and environmental factors, depicting convergences and complex interplays between genetic susceptibility and toxic insults. The pervasive nature of chemical exposure poses pivotal challenges for neurotoxicological studies, regulatory agencies, and policy makers. This highlights an emerging need of developing new integrative models, including biomonitoring, epidemiology, experimental, and computational tools, able to capture real-life scenarios encompassing the interaction between chronic exposure to mixture of substances and individuals’ genetic backgrounds. In this review, we address the intertwined roles of genetic lesions and environmental insults. Specifically, we outline the transformative potential of stem cell models, coupled with omics analytical approaches at increasingly single cell resolution, as converging tools to experimentally dissect the pathogenic mechanisms underlying neurodevelopmental disorders, as well as to improve developmental neurotoxicology risk assessment.

## Background

Neurodevelopmental disorders (NDDs) encompass a broad group of conditions characterized by alterations in the development of the central nervous system (CNS), resulting in varying degrees of cognitive and/or behavioral symptoms. Several conditions are grouped under the diagnosis of NDD, including intellectual disability (ID), learning disorders, communication disorders, motor disorders, attention deficit hyperactivity disorder (ADHD), autism spectrum disorder (ASD), epileptic encephalopathies (EE), and schizophrenia (SZ) [1, 2].

The worldwide prevalence of NDDs is estimated to be between 1 and 4% according to the systematic analysis of the Global Burden of Disease Study 2016 [3]. This value pools together intellectual disabilities, ASD, epilepsy, and ADHD.

NDDs entail a major global burden in terms of individual and family suffering, health care expenditure, and lost productivity [4].

Considering ASD, a systematic review on epidemiological surveys reported a global median of prevalence estimates of 62 per 10,000 [5]. In a report recently published by the Centers for Disease Control and Prevention, a surveillance conducted in several US states in 2016 identified an ASD prevalence of 18.5 per 1000 children aged 8 years (1 in 54); the prevalence was 4.3 times higher in boys than in girls. Among ASD children, 33% were classified as affected by intellectual disability, with higher frequency in girls than in boys (39% versus 32%) [6].

The heterogeneity of NDDs is further compounded by the increasing awareness of their blurred boundaries, both in terms of their clinical manifestations along the lifetime and the underlying multifactorial etiology and pathophysiology. One of the most important scientific promises and challenges of the next decade, indeed, is represented by the advance of precision psychiatric medicine in moving beyond the discovery of single factors that are associated with neuropsychiatric traits, towards the elucidation of the mechanistic relations that connect molecular mechanisms to clinical outcomes [7]. To address this challenge, a genotype-first approach has been advocated for the definition of ASD and more generally NDD molecular subtypes, as a way to overcome rigid diagnostic separations across disorders that are frequently overlapped (e.g., ASD and epilepsy) and at the same time to improve diagnosis and clinical management from a precision medicine perspective [8].

**Fig. 1:**
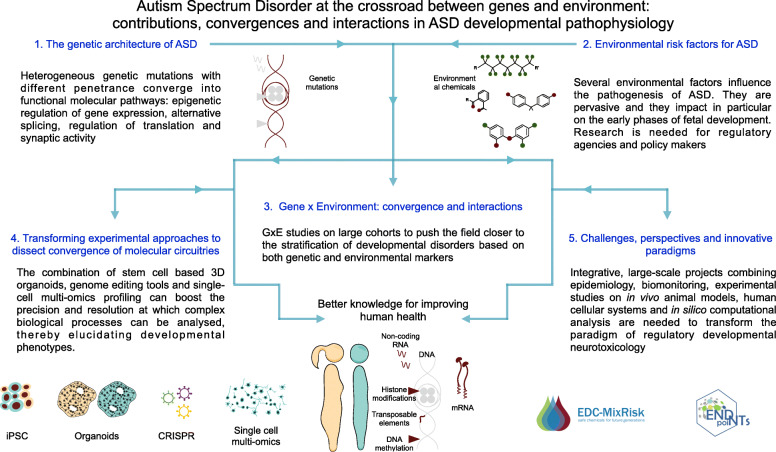
Autism Spectrum Disorder at the crossroad between genes and environment

Here, we will focus on ASD as a paradigmatic example of NDD whose manifestation is the outcome of a complex interaction of predisposition factors and genetic or environmental lesions. We will elucidate how both genetic alterations and toxic insults can be at the root of the pathogenetic events that trigger NDDs, highlighting the growing body of evidence pointing towards an interplay between the individual genetic make-up and the environmental exposures occurring in early life, and their convergence towards key molecular pathways. Finally, we will explore the groundbreaking experimental approaches that hold the promise to transform neurobiology and neurotoxicology by allowing the access and manipulation of neurodevelopmental key events at the single cell level, Fig. 1.

## The genetic architecture of ASD

Autism spectrum disorder defines a broad group of NDDs characterized by (i) young age of onset, (ii) impairment in communication and social abilities, (iii) restricted interests and repetitive behaviors, and (iv) symptoms that affect patients’ function in various areas of their life [2]. Symptoms’ severity varies widely and is often compounded by significant comorbidities, especially intellectual disability, epilepsy, anxiety, sleep, and gastrointestinal disorders [9, 10].

### Genetic causes of autism: rare and common genetic variants associated with ASD

Although a substantial genetic contribution to ASD is well recognized, its genetic architecture is exceedingly heterogeneous [11] and reflects a spectrum of genetic loads between two extremes: on the one hand a complex and still only poorly characterized burden of low-risk variants, mostly single nucleotide polymorphisms (SNPs), and on the other hand a large number of highly penetrant rare variants, often copy number variations (CNVs), whose expressivity is however also influenced by the heterogeneity of genetic backgrounds [12].

The first pattern of genetic variants contributing to ASD outlines a scenario involving a high number of SNPs, each of them conferring a small increase in the risk of ASD onset, with the hypothesis that clinically diagnosed ASD individuals can be considered as the extreme portion of a spectrum of genetically influenced behaviors that extend over the entire population. In agreement with this concept, common SNPs, distributed all over the genome, have been estimated to account for at least 20% of ASD liability and act additively or synergistically as risk factors [13–15]. For neuropsychiatric disorders that usually manifest later in life, such as schizophrenia, bipolar disorder, and major depression, genome-wide association studies (GWAS) have in fact elucidated a polygenic scenario with multiple alleles of very small effect [16–19]. Although most of the GWAS performed on ASD families also pointed to a similar outline, SNPs reaching genome-wide significance have remained elusive for a long period [20]. Only very recently, a large genotyping campaign carried out in the Danish population and comprising more than 30,000 individuals has yielded several genome-wide significant markers underlying 5 genetic loci [21]. This investigation also highlighted a substantial overlap between genetic variants associated with ASD and other neuropsychiatric disorders, pointing towards converging pathogenetic mechanisms. Finally, polygenic risk scores encompassing ASD, schizophrenia, and years of educational attainment have been recently shown to be unambiguously associated with ASD and to also contribute to the phenotype in the cases of patients carrying de novo deleterious mutations [16]. The second pattern of genetic variants contributing to ASD reflects the effect of rare, highly penetrant mutations or CNVs that are inherited or, more frequently, arise de novo in the germinal cells; being affected by negative selection because of their detrimental impact on reproductive fitness, their frequency in the general population remains very low [22]. Given the described difficulties in pinpointing common variants related to its onset, progress in ASD genetic research has so far advanced mainly with the discovery of rare, de novo, germline, coding, heterozygous mutations. These genetic alterations that are commonly observed in global developmental delay in addition to autism [23] contribute to about 20–30% of clinical cases of ASD [24–26] and offer transformative potential to illuminate ASD pathophysiology and new therapeutic routes [27].

Several large consortia have been established with the purpose to investigate the genetic causes of NDDs by genotyping large cohorts of patients. This has resulted in the generation of knowledge bases and repositories openly available online that represent valuable resources to explore the genetic cause of NDDs; the most relevant of them are listed in Table 1.

**Table 1:** Knowledge bases for NDD-relevant genes and patient cohorts

Resource	Link	Description
SFARI	https://gene.sfari.org/	913 genes cataloged and scored for their strength of association to ASD.
Autism KB	http://db.cbi.pku.edu.cn/autismkb_v2/	1379 syndromic and non-syndromic autism-related genes; 5420 CNVs or structural variations (SVs), 11,669 single-nucleotide variations (SNVs)/insertions and deletions (InDels), and 172 linkage regions associated with ASD.
DECIPHER	https://decipher.sanger.ac.uk	DDD study: data from about 14,000 children with severe undiagnosed developmental disorders and their parents.
MSSNG	https://research.mss.ng/	Whole genome sequencing from Autism Genetic Research Exchange (AGRE) families (about 10,000 individuals).
iPSYCH	https://ipsych.dk/en/research/	Danish national project examining in more than 130,000 subject risk factors for autism, ADHD, schizophrenia, bipolar disorder, and depression.

### Convergence of ASD risk genes in molecular functional domains

The complexity of ASD genetic architecture has been inspiring a sustained effort towards the identification of convergent molecular pathways that can group genetic mutations into categories sharing an analogous impact on neurodevelopment, thus explaining the similarity of the observed phenotypes. In fact, several studies are starting to indicate that, despite the pronounced heterogeneity emerging from a gene-centric view of ASD genetic causes, a higher degree of convergence becomes apparent when focusing on affected molecular processes and pathways. Specifically, the main emerging pathways are represented by epigenetic regulation of gene expression, alternative splicing, regulation of translation, and synaptic activity [7, 28–31].

The evidence of epigenetic and transcriptional dynamics as one of the functional domains more consistently linked to ASD is related especially to genes coding for DNA and RNA binding proteins [7]. Examples of key regulators of chromatin remodeling whose link with ASD is well established are CHD8 and CHD2 (both ASD high-risk genes), CTNBB1 (together with CHD8 acting on the WNT pathway), MECP2, and HDAC4 [10]. Among the transcription factors, TBR1 and FOXP1 have been shown to play a pivotal role in neurodevelopment [32, 33]. A paradigmatic example of an RNA-binding protein related to mRNA dynamics is FMRP (Fragile X mental retardation protein). Regarding synaptic functionality, affected genes are retrieved among key players of both glutamatergic and GABAergic transmission as well as scaffolding proteins such as those belonging to the SHANK family and neurexin cell adhesion molecules [10, 28, 34].

Even more interestingly, recent data indicates that this convergence at the functional level could hold true also when examining different NDDs and psychiatric disorders. In fact, mutations in chromatin regulators and transcription factors figure prominently among the most common genetic causes of NDDs, with more than 150 of them listed in the Simons Foundation Autism Risk Initiative (SFARI) database. This constitutes a first step to elucidate the broader phenotypic links between multiple NDDs, underlining the importance of identifying shared gene regulatory networks to better shape the classification of brain disorders and develop new therapeutic potentials [35]. In the same conceptual framework, fully penetrant NDD mutations in genes that regulate transcription appear to be particularly relevant as they operate at the regulatory control level, thus affecting cell fate and hence translating into developmental phenotypes. For example, the transcription factor GTF2I, located in the genomic region affected by copy number variation in Williams-Beuren (WBS) and 7Dup syndromes, is thought to contribute to the cognitive and social symptoms of both disorders [36]; GTF2I has been reported as a key contributor to the transcriptional dysregulation observed in the induced pluripotent stem cells (iPSC) derived from WBS and 7Dup patients [37]. In mice, GTF2I deletion in forebrain excitatory neurons has been associated with behavioral alterations such as increased sociability and anxiety [38]. YY1 is another transcription factor and epigenetic regulator that plays a crucial role in neurodevelopment, and whose haploinsufficiency has been associated with NDDs [39].

Finally, with GWAS performed on large cohorts, increasing evidence is pointing towards common genetic variants that are shared across several NDDs and psychiatric conditions, such as bipolar disorder and major depressive disorder, resulting in overlapping dysregulations and thus suggesting a common neuropathological architecture [40, 41]. This effort can be summarized by the comprehensive Psychiatric Cell Map Initiative, whose goal is to uncover new molecular, cellular, and circuit level understanding of neuropsychiatric disorders to reveal new targets for future therapies and bridge the gap between gene discovery and translational biology [4].

## Environmental risk factors for autism

In addition to the complexity of the genetic architecture underlying ASD, this spectrum of disorders is distinguished by the concomitant contribution of several environmental factors with an influence on its pathogenesis. Given its pervasive nature and its ability to influence the early phases of fetal development, the potential effect of environmental chemical exposure is gaining increasing attention [42]. However, an accurate and exhaustive estimation of the burden related to chemical substances on the manifestation of NDDs is particularly challenging considering that the current real-life scenario encompasses long-term exposures to a complex mix of substances that could interact in intricate ways among themselves and with individual genetic factors. This also results in the difficulty in establishing causality links between environmental exposure and clinical manifestations. As a consequence, while for some chemicals most of the epidemiological studies are convergent, in other cases, the findings are more conflicting and variable across examined cohorts. In this section, we aim at a panoramic view of the most relevant environmental factors that, together with the genetic components already discussed, have been implicated or suspected to contribute to the manifestation of NDDs. Although here we are not undertaking a systematic review of the body of literature on the topic, we have summarized the most relevant studies and their findings in Table 2, where we also reference to systematic efforts for most of the chemical classes under scrutiny.

**Table 2:** Systematic reviews and epidemiological studies focused on the association between chemical exposure and autism spectrum disorder

Study design	Main findings	Reference
**Heavy metals**		
Systematic review on ASD and child exposure to heavy metals.	Collectively, the studies support evidence of a positive association between exposure and ASD risk.	[43]
Cohort of monozygotic and dizygotic twins discordant for ASD; tooth-matrix biomarkers to estimate pre- and post-natal exposure to metals.	Higher levels of lead and lower levels of manganese in ASD. Moderate negative and positive association between respectively manganese and lead levels and ASD severity.	[44]
***Lead***		
Comparison between children with high and low concentration of lead in the blood.	Association of higher lead levels with decreased IQ performance.	[45]
Measurement of blood lead concentration in 172 children at several time-points.	Blood lead concentration inversely and significantly associated with IQ.	[46]
Pooled analysis on 1333 children from seven cohort studies.	Inverse relationship between blood lead concentration and IQ score, with 6.9 IQ point decrement (CI 4.2–9.4) for an increase in blood lead levels from 2.4 to 30 μg/dL.	[47]
***Methylmercury***		
Cohort of children in Faroe Islands followed from birth to 7 years of age. Mercury exposure measured in several biological matrices.	Association of exposure with language, attention, and memory dysfunctions.	[48]
Cohort of children in Faroe Islands followed from birth to 7 years of age. Mercury exposure measured in several biological matrices.	Prenatal methylmercury exposure is a predictor of neurobehavioral deficits.	[49]
Cohort of children in Faroe Islands.	Cohort members examined at 22 years of age: association between prenatal exposure and cognitive deficits.	[50]
Cohort of children from New Zealand; mercury levels measured in mother’s hair.	Association between high prenatal methylmercury exposure and decreased performance on psychological and scholastic tests.	[51]
Cohort of mother-child pairs from Seychelles. Mercury exposure measured in mother’s hair.	No relevant association found with several neurocognitive and behavioral functions in children at 9 years of age.	[52]
Systematic review on methylmercury prenatal exposure and neurodevelopmental effects.	Definition of a “lowest observable adverse effect hair concentration” from evidence by 48 studies on neurodevelopmental risks associated to methylmercury exposure.	[53]
**Air pollution**		
Systematic review on environmental toxicants and ASD.	Evidence from exposure to air pollution during gestation or childhood overall support an association with increased ASD risk.	[43]
Review on potential confounding factors in assessing association of air pollution and ASD.	General consistency of findings pointing to a causal association between air pollution and ASD.	[54]
Meta-analysis on 25 studies examining maternal exposure to air pollution and ASD risk.	Evidence of positive association for PM2.5, weak evidence for NO_2_, and little evidence for PM10 and ozone.	[55]
Meta-analysis on 23 studies examining developmental exposure to air pollution and ASD diagnosis.	Statistically significant summary OR for 10 μg/m^3^ increase in PM10 (OR 1.07; CI 1.06–1.08) and PM2.5 (OR 2.32; CI 2.15–2.51).	[56]
ASD prevalence and exposure to traffic-related air pollution assessed in a pregnancy cohort in Los Angeles County (California). 7603 ASD cases and 10 matched controls for each case.	12–15% relative increase in autism odds for each interquartile range (IQR) increase for ozone and PM 2.5.	[57]
Cohort of 645 children with autism born in North Carolina and 334 children born in San Francisco Bay area. Compared to 14,666 randomly sampled children born in the same county and year. Exposure to PM10 assessed by geostatistical interpolation methods.	Temporal patterns show an inverse correlation between PM10 concentrations in first and third trimester. OR estimated for 10 μg/m^3^ increase in PM10 after accounting for the correlation are 1.01 (CI 0.81–1.27) for the first trimester and 1.38 (CI 1.03–1.84) for the third trimester.	[58]
Nested case-control study of participants in the Nurses’ Health Study II. PM 2.5 and PM 10–2.5 exposure predicted from a spatio-temporal model.	Increased ASD risk for PM2.5 exposure, with OR per IQR increase of 1.57 (95% CI 1.22–2.03). Stronger association for exposure in the third trimester (OR 1.42; 95% CI 1.09–1.86).	[59]
Case-control study from southwestern Pennsylvania: 217 ASD cases compared to two control groups (intertwined controls and controls from random selection of birth certificates).	Analysis performed comparing fourth to first exposure quartile. Increased ASD risk identified for styrene and chromium; borderline effects for PAH and methylene chloride.	[60]
Case-control study on 279 ASD and 245 TD children from the Childhood Autism Risks from Genetics and the Environment study in California.	ASD children are more likely to live in areas with the highest quartile of traffic-related air pollution during gestation (OR 1.98; CI 1.20–3.31) and during the first year of life (OR 3.10; CI 1.76–5.57). Positive association with ASD found for nitrogen dioxide, PM2.5, PM10.	[61]
Cohort of children born in Los Angeles County (California) between 1995 and 2006 from mothers that resided nearby toxic monitoring stations.	Increase in ASD risk per IQR increase in average concentration for 1,3-butadiene (OR 1.59; CI 1.18–2.15), meta/para-xylene (OR 1.51; CI 1.26–1.82), lead (OR 1.49; CI 1.23–1.81), perchloroethylene (OR 1.40; CI 1.09–1.80) and formaldehyde (OR 1.34; CI 1.17–1.52).	[62]
CHARGE study: examined association between ASD and proximity of residence to freeways or major roadways during pregnancy for 304 ASD and 259 TD children.	Maternal residence in the third trimester (OR 2.22, CI 1.16–4.42) and at delivery (OR 1.86, CI 1.04–3.45) more likely near a freeway for ASD than TD.	[63]
ESCAPE project: 8079 children from several European birth/child cohorts.	No association identified for exposure to NO_2_ or PM with autistic traits.	[64]
Children from the Nurses’ Health Study II (325 cases, 22,101 controls). Exposure to air pollutants assessed at the time and place of birth from the US EPA-modeled levels.	Comparing highest versus lowest quintile, association with ASD reported for diesel, lead, manganese, mercury, and methylene chloride with OR from 1.5 to 2. A stronger association was found in boys compared to girls.	[65]
Retrospective cohort study on 246,420 children born in South California.	Identified a boy-specific association between PM2.5 levels and ASD risk (hazard ratio for first trimester exposure 1.18 per 6.5 μg/m^3^; CI 1.08–1.27).	[66]
Population-based cohort of 132,256 births in Vancouver; prenatal exposure to PM2.5, NO, and NO_2_ estimated.	Association between exposure to NO and ASD (OR for IQR increase 1.07; CI 1.01–1.13).	[67]
Case-control study on 124 ASD and 1240 TD from Shanghai. Exposure to PM1, PM2.5, and PM10 estimated for the first 3 years after birth.	Association with increased ASD risk for an IQR increase for PM1 (OR 1.85, 95% CI 1.09–3.17), PM2.5 (OR 1.78, 95% CI 1.14–2.75), and PM10 (OR 1.68, 95% CI 1.09–2.59).	[68]
**Endocrine disruptors**		
Review on the links between endocrine disruptors and neurodevelopment.	Converging body of research from animal models, clinical observations, and human population studies implicates EDCs in an array of neurodevelopmental disorders.	[69]
Review summarizing epidemiological studies on the relations of early-life exposure to bisphenol A (BPA), phthalates, triclosan, and perfluoroalkyl substance (PFAS) with childhood neurobehavioral disorders and obesity.	Prenatal exposure to several EDCs is associated with adverse neurobehavior (BPA and phthalates) and excess adiposity or increased risk of obesity/overweight (PFAS).	[70]
***Phthalates***		
Prospective cohort study of primiparous women in New York between 1998 and 2002 (*n* = 404). Third-trimester maternal urines analyzed for phthalate metabolites. Children (*n* = 188, *n* = 365 visits) were assessed for cognitive and behavioral development between the ages of 4 and 9 years.	Behavioral domains adversely associated with prenatal exposure to low molecular weight phthalates.	[71]
Prospective cohort study in New York. Mono-n-butyl phthalate, monobenzyl phthalate, monoisobutyl phthalate, and four di-2-ethylhexyl phthalate metabolites measured in urine. 319 women sampled in the third trimester. Mental, motor, and behavioral development in children at 3 years of age.	Prenatal exposure to Di-n-butyl phthalate (DnBP), diisobutyl phthalate (DiBP), and benzyl butyl phthalate (BBzP) may adversely affect child mental, motor, and behavioral development during the preschool years.	[72]
Australian pregnancy cohort of 1064 women; exposure to phthalates measured in maternal urine. Oxidative stress-related genetic score calculated from a panel of SNPs.	Higher exposure to phthalates was associated to ASD (OR 1.65 per SD unit increase in phthalates; CI 1.00–2.72). Multiple phthalate-SNP interactions observed.	[73]
***Bisphenol A***		
Prospective cohort study in Cincinnati (2003–2006). BPA concentrations at 16 and 26 weeks gestation (*n* = 389). Thyroid stimulating hormone (TSH) and free and total thyroxine (T4) and triiodothyronine (T3) at 16 weeks (*n* = 181).	Prenatal BPA exposure may reduce TSH among newborn girls, particularly when exposure occurs later in gestation.	[74]
Collaborative project called the Consortium Linking Academic and Regulatory Insights on Toxicity of BPA (CLARITY–BPA) launched by three US federal agencies: the FDA, the NIH National Institute of Environmental Health Sciences, and the National Toxicology Program.	Published studies indicate among the most consistent effects those of BPA on the brain, including alterations of the volume of sexually dimorphic structures and gene expression within specific brain regions.	[75]
***Perfluoroalkyl substances***		
Scientific Opinion of the European Food Safety Authority (EFSA) panel on contaminants in the food chain.	Administration of PFOS to 10-day old mice has been reported to result in impaired performance in behavioral tests conducted when the mice were 2 and 4 months old.	[76]
A nested case-control study in the Danish National Birth Cohort (1996–2002). 220 cases of ADHD, 220 cases of ASD, 550 controls. Sixteen PFASs were measured in maternal plasma collected in early or mid-pregnancy.	No consistent evidence to suggest that prenatal PFAS exposure increases the risk of ADHD or childhood autism in children.	[77]
A matched case-control study in Malmö, Sweden (1978–2005). Children with ADHD (*n* = 206), controls (*n* = 206). PFOS and PFOA concentrations were measured in umbilical cord serum samples.	The study revealed no support for an association between fetal exposure to PFOS, PFOA, or PFNA and ADHD.	[78]
***Polychlorinated biphenyls***		
Mother-child pairs from MARBLES (California, Davis, from 2006). PCB concentrations were measured in maternal blood at each trimester. Clinical diagnosis of ASD and non-typical development compared to typically developing (TD) analyzed in 3-year-old children.	This study does not provide strong supporting evidence that PCBs are risk factors for ASD or non-typical development.	[79]

*CI* 95% confidence interval, *OR* odds ratio*, IQR* interquartile range*, SD* standard deviation, *PM2.5* particulate matter ≤ 2.5 micron*, PM10* particulate matter ≤ 10 micron

### The specific vulnerability of the developing brain

The developing CNS is particularly vulnerable to external insults. This is related on the one hand to the highly complex, specific, and coordinated series of biological events guiding early human brain development and on the other hand to the lack or incomplete functionality of barriers such as the blood brain barrier. It is therefore not surprising that the gestational and perinatal periods are recognized as windows of vulnerability in neurotoxicology; in fact, a variety of external perturbations in prenatal and perinatal periods have been widely reported in association with ASD [80] and more in general with NDDs.

Among the environmental factors that have an impact on ASD onset and act in the developmental phases, there are several maternal-related determinants including maternal nutrition, hormonal equilibrium, and stress status, as well as substance abuse and exposure to environmental chemicals, including air pollutants, pesticides, plastics derivatives, and metals [81, 82]. Prenatal exposure to alcohol is one example of a non-genetic risk factor, hypothesized to act through the dysregulation of the SHH pathway. In a study conducted on a representative Midwestern US community, the prevalence of fetal alcohol syndrome was estimated from 6 to 9 per 1000 children, and the rate of fetal alcohol spectrum disorders was 2.4 to 4.8% [83]. Also, many studies observed a higher risk for ASD or ADHD symptoms in subjects prenatally exposed to tobacco smoke [84]. Maternal nutrition is crucial to ensure the correct fetal supply of nutrients, in particular for fat-soluble vitamins (A, D, E), tryptophan, and nutrients related to single carbon metabolism (choline, vitamins B2, B6, B12, and folate) known to contribute to the methylation of metastable epialleles in the progeny that is persistent also in differentiated tissues [85]. The maternal stress status, acting through the neuroendocrine axis, has also been associated with alterations of the epigenetic programming that can impair correct neuronal development [86]. As a paradigmatic example, evidence from rodent studies also indicates maternal care deprivation as impacting on the function of progeny’s brains by modulating the activity of the retrotransposon LINE-1 (a key source of somatic mosaicism in the brain) as well as by altering the methylation of YY1 binding sites and the expression of the DNA methyltransferase DNMT3A [87]. As for drug use, epidemiological studies showed that prenatal exposure to SSRI (selective serotonin reuptake inhibitors) [88] and valproic acid [89] increases the risk of ASD in the offspring, and even prenatal exposure to paracetamol has an adverse impact on language development [90]; however, the current literature evidences have not yet elucidated how specific drugs can increase the risk of ASD, highlighting the need of further research on this topic.

While some of the maternal-associated factors, such as stress and care deprivation, are difficult to examine quantitatively, efforts are ongoing to tackle the challenging task to precisely follow the exposure to chemical substances in gestational and mother-child cohorts.

From a historical point of view, very frequently the attention on the impact of such chemicals on the CNS was drawn by obvious effects ensuing exposure to high doses in both adults and children; this then led to the investigation of subtler but more pervasive effects at lower doses especially on the developing brain [91].

### Heavy metals

Exposure to heavy metals such as mercury, lead, and arsenic has been robustly associated with neurodevelopmental disorders [91, 92] as well as to adverse neurodevelopmental outcomes linked to ASD [93]. Both epidemiological data and animal studies have highlighted an association between exposure to lead and neurodevelopmental alterations [46, 94, 95]. The proposed molecular mechanisms are diverse, ranging from imbalances in calcium homeostasis [96, 97], impaired synaptic functionality [98], and alteration of the blood brain barrier [99].

Methylmercury neurodevelopmental toxicity, mainly due to contaminated seafood, is widely reported [92], with pathogenetic mechanisms related to oxidative stress and altered calcium and glutamate homeostasis [100]. Beyond very apparent effects registered after exposure at high doses, there is evidence underlying developmental neurotoxicity also for lower doses [101]. Three major epidemiological studies have examined the association between pregnancy low level exposure and neurobehavioral deficits in children in the Faroe Islands, New Zealand, and the Seychelles. Two of them have highlighted an association between maternal levels and subtle neurobehavioral deficits [48, 51, 92], with indications of defects persisting in adult life [50]. In contrast, the Seychelles study did not find an association after correcting for post-natal exposure [52]. In children, exposure to methylmercury has been associated with neurobehavioral alterations [102].

Focusing on ASD prevalence, epidemiological data, although in some cases limited by study design and sample size, overall point towards an association of gestational and post-natal exposure to several heavy metals [43]. A recent study, estimating from deciduous tooth matrix gestational and early post-natal exposure to metals in monozygotic and dizygotic twins discordant for ASD, revealed differences in metal uptake affecting specific developmental windows [44].

### Air pollution

Air pollutants such as gasses, particulate matter, metals, and chemicals have been reported by the Environmental Protection Agency to be hazardous to human health [103]. While so far the focus of the research in this field has been on respiratory and cardiovascular diseases, both in vitro and epidemiological studies are mostly concordant in indicating effects of air pollution also on the brain. Studies in animal models exposed to air pollutants have highlighted a complex range of effects on the CNS, depending on both the examined model and the exposure paradigm, with mechanisms related to oxidative stress and neuroinflammation as those described more frequently [104–106]. An increasing body of epidemiological studies suggest pre-natal and post-natal exposure to air pollution as a potential risk factor for ASD [43, 57–63]. Although this is not confirmed by all the gathered evidences [64, 67], the majority of the studies are largely consistent in reporting an association also after taking into account possible confounders [54]. A sex-specific interaction has also been suggested, with boys being more susceptible than girls [65, 66].

### Endocrine disruptors

An endocrine-disrupting chemical (EDC) is defined by the World Health Organization as an exogenous substance or mixture that alters function(s) of the endocrine system and consequently causes adverse effects in an intact organism, or its progeny, or (sub)population [107]. The definition of EDC is a non-trivial process, since it entails complex mechanisms that have to be considered and has vast impacts on regulatory aspects and therefore public health. The relevance for a high number of stakeholders is also shown by the recent effort of the European Commission in mediating between industry, society, institutions, and scientific results to define EDC and the policies for their employment [108]. As an example, it is still not clear if heavy metals such as lead, cadmium, mercury, arsenic, manganese, and zinc that are known neurotoxicants should be classified as EDCs. EDCs impact on fetal CNS is of particular concern because, through the dysregulation of the delicate hormonal balance, epigenetic responses can mediate effects on neural progenitors resulting in long-term adverse health impact [69, 84, 109]. EDC adverse effects on neurodevelopment, indeed, can act at different scales and times, such as progenitor proliferation and migration, as well as neuronal maturation or the synthesis, transportation, and release of neurotransmitters [69]. EDC association to ASD has been hypothesized to be mediated by the alteration of different hormonal signaling pathways as well as epigenetic modifications that need to be systematically characterized yet [84, 110]. In the following paragraph, the endocrine-disrupting compounds that are better known in terms of their molecular effect on neurodevelopment are reviewed.

Phthalates derive from a multitude of consumer products, including personal care products, medications, and plastics. They are ingested, inhaled, and absorbed from derma, and they can also cross the placenta [111–113]. Because of their chemical properties, they may interfere with the action or metabolism of androgens, thyroid hormones, and glucocorticoids. Prospective cohort studies have associated phthalates with ADHD and ASD, reduced mental and psychomotor development, emotional problems, and reduced IQ [71, 72].

Bisphenol A (BPA) is incorporated in several plastics that are present in daily life consumer products. It is known to act through the binding of estrogen receptors α and β as a weak agonist [114], but it can also interfere with androgen and thyroid signaling pathways [74]. Different studies suggest that BPA exposure is associated with behavioral and cognitive problems in children, but there are inconsistencies with regard to the period of life with the greatest vulnerability and sex-specific effects [70, 75]. Very recently, it has also been suggested that, due to technical limitations in biomonitoring measurements, human exposure to BPA could have been importantly underestimated [115].

Perfluoroalkyl substances (PFAS) are used in water-resistant materials, industrial surfactant, and food containers, thus, like phthalates and bisphenols, are ubiquitously found in the environment [76]; they are very persistent and as such prone to bioaccumulation. They are active on several endocrine axes, in particular interacting with the peroxisome proliferator activated receptor (PPAR), glucocorticoid, and thyroid pathways [116–118]. Exposure to PFAS however has not been linked to ASD and ADHD risk by epidemiological studies [77, 78].

Polychlorinated biphenyls (PCBs) were widely employed industrially as coolants, plasticizers, and flame retardants. Although their production was banned, given their resistance to degradation, their presence in the environment persists. PCB mechanisms of action are not well-known, and the association between exposure and inattention, impulsiveness, and other ADHD-related behaviors needs yet to be confirmed by further studies [79].

Finally, neurotoxicological studies on EDCs have brought to the attention of scientists and regulators three important issues that are usually underestimated and, in general, not properly addressed in other contexts: (i) the impact of low doses, (ii) long-term effects, and (iii) interactions occurring because of mixtures of compounds. It was observed, indeed, that EDC doses similar to environmental levels can have significant effects in experimental models, showing peculiar dose-response patterns that are not monotonic [119], even if this phenomenon has yet to be validated in other settings [120]. Moreover, EDCs can cause effects that only manifest much later in life than the period of exposure [121], most likely because of epigenetic mechanisms of information transmission: the exposure to the chemical could result in epigenetic modifications of the genome that can be inherited by future generations [122]. As far as EDC mixtures are concerned, there are still huge gaps of knowledge regarding their mechanisms of action, especially if considering molecules that interfere with different hormonal pathways, for which additive predictive models are not suited and additional experimental evidence and testing systems are required [123, 124]. To conclude, EDCs are posing pivotal questions regarding their toxicological and regulatory profile, drawing the attention on the need to find innovative tools and models to study and regulate the potential danger associated with chemical exposure; they thus embody both the challenge and the opportunity to translate science into policies [125].

## Gene × environment: convergence and interactions

### Interactions between genetic and environmental causes of ASD

The topics discussed so far underline the complex interplay between genetic and environmental factors in ASD pathophysiology. This is further shown by different studies that are trying to address the multifaceted etiology of ASD examining the combination of the two factors at the same time. This effort is highlighting not only that both genes and environment can cause ASD, but also that dissecting their interactions is crucial to understand the contribution of the genetic background to environmental stressor response.

One of the mechanisms by which genes and environment interact in ASD pathogenesis is related to polymorphisms in genes that regulate the response to endo- or xeno-biotics, such as the differential susceptibility to estrogen exposure in different mouse strains [126], or by the evidence of SNPs that in mice confer resistance to the adverse effects induced by the endocrine disruptor Di(2-ethylhexyl)phthalate (DEHP). In the latter case, in particular, the effect is mediated by the increased expression of the estrogen receptor as well as by the altered methylation of specific promoters, regulated in opposite direction by the genetic variants and the chemical exposure [127]. Interactions between genetic and environmental factors in determining social and cognitive outcomes have also been reported in rodent models carrying a specific *Mecp2* truncating mutation, a condition that provides resistance to polybrominated diphenyl ethers effects on short memory [128].

In humans, studies examining the influence of specific genetic variants on the susceptibility to toxicants have identified SNPs in enzymes involved in xenobiotic metabolism such as paraoxonase 1 (PON1), glutathione-S-transferases (GSTM1 and GSTP1), δ-aminolevulinic acid dehydratase (ALAD), solute carrier family 40 member 1 (SLC40A1), and the metal regulatory transcription factor 1 (MTF1) that were associated with ASD risk [43]. Other examples of studies that have tackled this issue revealed that (i) a genetic variant in the receptor tyrosine kinase (MET) contributes to increase the risk of ASD together with prenatal exposure to air pollutants [129]; and (ii) genetic variants in the one carbon metabolism pathway interact with the maternal use of prenatal vitamins for ASD risk, in a study where the pre-conceptional period and the first pregnancy trimester resulted as the most critical ones [130]. A recent investigation of an Australian pregnancy cohort of 1064 women has taken into consideration the role of prenatal exposure to phthalates and of genetic variants associated to oxidative stress (a candidate mechanism of phthalate toxicity) in ASD risk. Increased exposure to phthalates as well as increased oxidative stress-related genetic score were found associated to ASD; the co-occurrence of the two factors was identified as particularly detrimental, thus underlying the importance of considering genetic and environmental interplays [73].

A further level of complexity of gene × environment (G×E) interactions recently emerged from a study performed on a cohort of twins including typically developed (TD) individuals and ASD patients. This has shown, through the analysis of structural brain measures, that distinctive clinical phenotypes are influenced by genes and environment in a different way between patients and controls. As a matter of fact, on the one hand, genetic factors accounted for the majority of variation in brain size in both ASD patients and TDs, in particular as far as the curvature and subcortical gray matter were concerned. On the other hand, cortical thickness and cerebellar white matter volume were primarily influenced by environmental factors in ASD but not TD twin pairs [131]. Some environmental factors could also act by increasing the risk of DNA mutagenesis [132]; in fact, it has been hypothesized that exposure to heavy metals and vitamin D deficiency increase the frequency of de novo mutations in ASD-causing genes [133]. On the same line of evidence, a recent study has reported PCB-95, a polychlorinated biphenyl compound, as impacting CNV frequency at 15q11-q13 locus, linked to ASD [134].

Finally, another mechanism underlying the convergence between genetic predisposition and environmental exposure is the interference of the latter with the regulation of the expression of the genes involved in key molecular pathways often disrupted in NDDs. Valproic acid, for example, has an inhibitory effect on histone deacetylase, thus affecting the epigenetic landscape in a way that was revealed able to reverse symptoms in bipolar disorder patients [135]. Folate deficiency, instead, can have an impact on DNA methylation that, in turn, impairs the physiological neurodevelopment and results in adverse effects on mental health [136]. Our recent experiments performed on human neurodevelopmental models revealed that the specific mixture of EDCs that was associated to neurodevelopmental outcomes at the epidemiological level has a transcriptional impact on the same genes whose mutations are causative of ASD [137]. In summary, a growing body of evidence is accumulating indicating an interplay between genetic and environmental factors in ASD pathogenesis. Yet, the current knowledge about G×E interactions is still limited, and studies to systematically evaluate these mechanisms are hindered by the difficulties in retrieving high-quality exposure and genetic data for large cohorts of individuals, needed to further illuminate the most relevant interactions at the population level [138]. This is especially challenging for the G×E of low penetrant common variants, since most of the effort so far has been focusing in dissecting the impact of specific environmental factors on highly penetrant NDD mutations [139].

To conclude, the increasing resolution of environmental data records given by initiatives such as the Human Exposome Project [140], coupled with the growing number and depth of genomic profiles, will likely result in powerful and high-quality G×E studies on larger cohorts and thus push the field closer to the stratification of developmental disorders based on both genetic and environmental markers [138, 141, 142].

## Transforming experimental approaches to dissect convergence of molecular circuitries

### iPSC and brain organoids to dissect temporal and spatial dynamics in neurodevelopment

The possibility of deriving human pluripotent stem cells, either from embryos (embryonic stem cells, ESCs) or from somatic cells (induced pluripotent stem cells, iPSCs), has allowed in recent years to gain experimental access to biological systems and stages that have been so far elusive. The developing CNS is a paradigmatic example: the introduction of cell reprogramming technologies has transformed our abilities to model and study brain development. In fact, 3D brain organoids derived from iPSCs have increasingly emerged as a groundbreaking approach for overcoming the inaccessibility of the spatial and temporal dynamics of human fetal brain development, recapitulating its most salient features. As such, they can be exploited to dissect the cellular and molecular mechanisms at the basis of physiological human corticogenesis [143], as well as for the study of genetic and environmental perturbations affecting its trajectories. This is particularly relevant considering that the use of rodent models to scrutinize the pathogenesis of such complex brain disorders encounters an important limitation in the scarce representation of specific cell types, particularly those related to human cerebral cortex expansion [144].

Brain organoids are composed by the main cell populations characteristic of the fetal brain such as neural progenitors, neuronal, and glial population, organized in morphologically defined domains with progenitor cells distributed in the inner regions (ventricle-like structures) while maturing cells migrate outwards. The cellular composition varies according to the specific model [145]. In fact, several experimental protocols have been developed by research groups, falling in two main approaches: unpatterned and patterned protocols. In the first case, the emergence of developmental trajectories results from the intrinsic programs of cellular aggregates [146–149]. The second approach is based on the use of small molecules and growth factors applied to selectively guide the differentiation towards a specific brain region, such as the forebrain, the midbrain, the hippocampus, or the thalamus [150–153]. While unpatterned brain organoids are richer in terms of cellular and regional diversity, they are also less reproducible, showing higher variability across differentiation rounds compared to patterned protocols [154].

Brain organoids represent a promising approach to overcome the inherent constraints associated to 2D cultures or simpler 3D models by better capturing the physiological characteristics and developmental trajectories of their in vivo counterpart. The research in this field is active in devising improvements to current technical limitations, such as the generation of “vascularized” organoids to limit metabolic stress [155] and organizing centers to recapitulate morphogen gradients responsible for the anterior-posterior and dorsal-ventral axes [156].

A number of different brain disorders have been already modeled and manipulated by taking advantage of brain organoids, such as microcephaly related to CDK5RAP2 mutations [148], or considering an environmental insult, neurodevelopmental delays related to Zika virus exposure [157, 158]. Focusing on ASD, idiopathic cases have been studied by means of iPSC-derived brain organoids [159]: transcriptomic analysis revealed an imbalanced overproduction of GABAergic interneurons, hypothesized to be dependent on FOXG1 dysregulation. In a cohort of patients affected by ASD associated with macrocephaly, cerebral organoids, together with other experimental models, have revealed a perturbations of the physiological steps of neuron development, associated to an early stage pathological priming of molecular circuitries [160]. Timothy syndrome, a severe neurodevelopmental disorder with ASD symptoms due to mutations in CACNA1C gene, has also been studied through glutamatergic and GABAergic organoids “assembloids,” with results showing an impairment of inhibitory neuron migration [161]. Finally, a novel application for brain organoids is their use for in vitro chemical screening, as discussed in more detail in the section on “ASD and developmental neurotoxicology: priorities, challenges, perspectives, and innovative paradigms”.

### CRISPR genome editing to selectively target specific pathways and validate molecular hypothesis

Genome editing technologies based on CRISPR represent a ground-breaking advance to introduce genetic or epigenetic mutations and investigate their mechanism of action. Their application in biomedical research would allow to accomplish two main objectives: (i) the regeneration of healthy specialized cells, after correcting mutations, with the idea of transplanting them into the differentiated organs, and (ii) the interrogation of the mechanistic role of multiple genes in the course of hardly accessible physiological processes such as early developmental stages [162].

The use of CRISPR in combination with organoid differentiation has already been exploited in cancer research by several studies that either introduced or corrected genetic mutations, to then profile the phenotypic effects in intestinal and kidney organoids, as well as to study the differential sensitivity of multiple genetic backgrounds to several drugs [163–166].

Even if similar approaches have not been published yet in the context of brain organoids and neurodevelopmental disease modelling, the same principles illustrated above are valid, and the neuroscience community will benefit soon of the mechanistic dissection that genome editing on organoid can illuminate [167]. In particular, the emergence of experimental strategies that allow to multiplex the CRISPR perturbations in few samples are significantly increasing the number of individual genomic loci that can be profiled in the same experiment, and thus the reach of CRISPR screenings. Different approaches to perform the systematic perturbation of genomic or epigenomic loci at the same time are already available; for example, Datlinger and co-workers [168] pooled in the same population of cells thousands of different genomic edits, then reconstructed relying on barcodes that were introduced with the CRISPR library and on deconvolution algorithms that associate the effect of each perturbation to a gene expression readout. Moreover, the combination of different types of perturbations (activating and inhibitory) in the same populations in an orthogonal CRISPR screening allows to understand the direction of information flow for characterizing how genetic networks regulation is translated into phenotypes [169]. Finally, a recently developed strategy can be exploited to interrogate genomic regulatory regions by introducing random combinations of enhancer perturbations (based on dCas9-KRAB complex) in a population of cells that can be then profiled in a single round of experiments [170].

The impact of these experimental strategies on developmental neuroscience will be substantial, with the imminent application of CRISPR multiplexed perturbations on brain organoids shedding light on the precise contribution of multiple genomic regions to cellular phenotypes throughout the different stages of brain development. A first applicative example, although in a murine model, is the study of 35 ASD risk genes [171] in which in utero pooled genome editing of forebrain progenitors was followed by single cell transcriptional analysis at postnatal stages to uncover their effect in specific cell populations.

### scRNASeq to obtain a read out at the single cell level

Together with 3D brain organoids and genome editing tools, single-cell omics has the potential to critically boost the precision and resolution at which biological structures composed by heterogeneous and intertwined cellular populations can be analyzed. Single cell approaches aim at profiling the genome, transcriptome, histone modifications, chromatin accessibility, DNA methylation, or protein signatures of single cells, overcoming the inherent limitations associated with the averaged read-out of bulk techniques and thus gaining information on the molecular state of each cell that constitutes a complex tissue or organ. Among these techniques, single cell RNASeq (scRNASeq) is at the moment the most established and widely used. Based on this approach, large consortia like the Human Cell Atlas Project are trying to build reference maps of the molecular states of all the cell types in healthy human tissues to study physiological states, developmental trajectories, regulatory circuitries, and interactions, and also provide a framework for understanding cellular dysregulation occurring in pathological states [172–175]. From a developmental point of view, one of the most interesting potentials of single cell omics data analysis is the reconstruction of developmental trajectories. Single cell transcriptomes can be projected, after dimensionality reduction, in a common analytical space, through different algorithms that can define a distance between the single cells coming from a complex tissue, on the basis of the similarity of their transcriptomes, thus defining a new temporal concept defined as pseudotime [176, 177] and introducing the possibility to predict future developmental trajectories for each single cell through RNA-velocity [178–180].

Several studies have already taken advantage of these techniques to investigate the molecular mechanisms at the basis of the differentiation of heterogeneous cerebral cell populations. For example, Polioudakis and co-workers exploited scRNASeq to derive an atlas of the developing human cortex, defining cell types on the basis of their transcriptional signature and reconstructing related regulatory networks [181]. Other studies have extended scRNASeq also on brain organoids for the study of their transcriptional and epigenomic landscape [182] or to investigate characteristics and trajectories of brain development that are human specific compared to other primates [183].

Our recent work took advantage of scRNASeq and brain organoids to probe the impact of chronic GSK3 inhibition on neurogenesis, uncovering its effect on proliferation and differentiation dynamics of neural progenitor populations [143]. Finally, in a single cell RNASeq study performed on post-mortem cortical tissue of ASD patients, alterations were identified both in neural cells, with dysregulation of synaptic functionality in upper-layer neurons, and glial populations, with activation of microglia [184].

## ASD and developmental neurotoxicology: priorities, challenges, perspectives, and innovative paradigms

As this review has outlined, the etiology of neurodevelopmental disorders is frequently multifactorial and underlies a complex interplay of multiple genetic and environmental contributors. Even in monogenic forms, individual genetic background and environmental exposure can play a modifying role by influencing disease expressivity. In line with this, the overall increase in prevalence reported in recent years for NDDs, and especially ASD, has been suspected to be linked to increased exposure to environmental toxicants [185]. This presents crucial concerns and challenges from a toxicological and regulatory point of view. In this paragraph, we will discuss the most relevant ones, reporting as paradigmatic examples, efforts in which our as well as other research groups are involved to tackle them. For most of the thousands of anthropogenic chemicals that are present on the market and in the environment, the information about their developmental neurotoxicity (DNT) properties is limited, highlighting an extensive knowledge gap. In fact, both in EU and the USA, compounds are not routinely tested for developmental neurotoxicity, with DNT properties investigated only upon indications of neurotoxicity or endocrine interference observed in adult rodent organisms [186]. As a consequence, only few compounds have been tested for their DNT potential and have been recognized as toxicant during the neurodevelopmental window. This is particularly problematic when taking into consideration that, as already discussed, the developing CNS is largely recognized to be more vulnerable than the adult brain to chemical perturbations [91]. Taken together, these considerations highlight an urgent need for a new framework for the assessment of chemicals with potential neurodevelopmental toxicity [185]. To tackle this issue, a key component is the setup of a battery of complementary, easy-to-standardize, and cost-effective high-throughput in vitro screening assays, able as an ensemble to recapitulate as faithfully as possible the complex events of human neurodevelopment, and therefore to overcome the limitations of in vivo rodent models related to inter-species differences in brain morphology and functionality. An example of this innovative approach is being developed by the ENDpoiNTs consortium [187], whose purpose is to advance the scientific knowledge on how EDCs exert their negative effects on neurodevelopment while also developing new screening tools for this class of compounds. From a mechanistic perspective, increasing the understanding on the mode of action and key molecular events that are triggered by toxicants would impact on our knowledge of the fundamental biological processes that, when perturbed, alter the physiological trajectories of neurodevelopment, thus adding valuably on our insight on NDD pathogenetic events and convergences.

In the context of the setup of in vitro assays tailored to relevant key events of human neurodevelopment, 3D models such as brain organoids are particularly promising: coupled with single cell omics readouts, they allow to pinpoint the effect of chemical perturbations on different players and developmental trajectories, taking into account complex interactions among cell types but also breaking down the observed effects on specific populations. This brings also the advantage to widen the focus of DNT research that is currently mostly neuron-centric, to other, less examined cell types of the developing CNS. The possibility to derive organoids from different iPSC lines, either separately or in combination in a single organoid (multiplexed organoids) then deconvoluted by in silico approaches [188–190] is instrumental to dissect the impact of a toxic insult on different human genetic backgrounds.

Another emergent challenge in developmental neurotoxicology is the need to take into account both in epidemiological and regulatory settings real-life scenarios involving chronic exposure to multiple chemicals. To tackle this challenge, perspective studies based on large cohorts followed for long periods of time are needed. In the innovative concept of exposome, this is coupled with the assessment of environmental exposure. Among the efforts active in this field, the EU-funded Human Early Life Exposome [191] monitors exposure in pregnancy and early life to evaluate the impact on child health outcomes. Another EU-funded project, NEUROSOME [192], is tackling the association between NDDs and cumulative early life exposure to chemicals such as metals and persistent organic compounds, taking into consideration also the contribution of genetic predisposition. A second key point is the need to shift from a “single compound” to a “mixture” paradigm. In fact, evidence about mixture effects in long-term exposure is accumulating [123], but still risk assessment is mostly focused on single substances. This can lead to a substantial underestimation of the risks when effects are additive or synergistic. EDC-MixRisk [193] is an EU Horizon 2020 research project that examined the effects of prenatal exposure to mixture of chemicals with suspected EDC activity on adverse effects in children, with the ambition to study the mechanistic aspects as well as to provide tools and insight for risk assessment. The multi-disciplinary approach has combined epidemiology and experimental biology, employing complementary in vivo and in vitro models. The employed strategy has harnessed brain cortical organoids, in parallel with human fetal primary cells, as well as in vivo models, to study the molecular mechanisms by which the real-life environmental concentrations of EDCs, measured and studied in the SELMA epidemiological study, can affect human neurodevelopment [137], thus introducing a transforming paradigm for regulatory neurotoxicology.

## Conclusions

In conclusion, neurodevelopmental disorders are the results of the contribution and interplays between the individual genetic makeup and the environment in which the organism develops and grows, with windows of vulnerabilities in prenatal and early postnatal phases. Groundbreaking experimental approaches such as iPSC-based brain organoids, single cell omics, and genome editing techniques show great promise to advance our understanding of the molecular conduits causally linking both genetic vulnerabilities and environmental exposures to NDDs. This will be instrumental for neurobiology to elucidate the pathogenic mechanisms at the basis of these disorders and for neurotoxicology to improve risk assessment, with an arguably major impact on public health in terms of actionability for lifelong conditions of such significance.

## Data Availability

Not applicable.

## Abbreviations

ADHD: Attention deficit hyperactivity disorder
AGRE: Autism Genetic Research Exchange
ASD: Autism spectrum disorder
BPA: Bisphenol A
CI: 95% confidence interval
CNS: Central nervous system
CNVs: Copy number variations
DEHP: Di(2-ethylhexyl)phthalate
DNT: Developmental neurotoxicology
EE: Epileptic encephalopathy
EDC: Endocrine-disrupting chemicals
ESCs: Embryonic stem cells
G×E: Gene × environment
GWAS: Genome-wide association studies ID Intellectual disability
iPSC: Induced pluripotent stem cells
IQR: Interquartile range
NDDs: Neurodevelopmental disorders
OR: Odds ratio
PCBs: Polychlorinated biphenyls
PFAS: Perfluoroalkyl substances
PM2.5: Particulate matter ≤ 2.5 micron
PM10: Particulate matter ≤ 10 micron
PPAR: Peroxisome proliferator activated receptor scRNASeq Single cell RNA sequencing
SD: Standard deviation
SFARI: Simons Foundation Autism Risk Initiative
SNPs: Single nucleotide polymorphisms
SSRI: Selective serotonin reuptake inhibitors
SVs: Structural variations
SZ: Schizophrenia
TD: Typically developed
WBS: Williams-Beuren Syndrome
